# Information-theoretic analysis of realistic odor plumes: What cues are useful for determining location?

**DOI:** 10.1371/journal.pcbi.1006275

**Published:** 2018-07-10

**Authors:** Sebastian D. Boie, Erin G. Connor, Margaret McHugh, Katherine I. Nagel, G. Bard Ermentrout, John P. Crimaldi, Jonathan D. Victor

**Affiliations:** 1 Feil Family Brain and Mind Research Institute, Weill Cornell Medical College, New York, New York, United States of America; 2 Civil, Environmental, and Architectural Engineering, University of Colorado, Boulder, Colorado, United States of America; 3 Department of Neuroscience & Physiology, NYU Langone Medical Center, New York, New York, United States of America; 4 Department of Mathematics, University of Pittsburgh, Pittsburgh, Pennsylvania, United States of America; University of California Santa Barbara, UNITED STATES

## Abstract

Many species rely on olfaction to navigate towards food sources or mates. Olfactory navigation is a challenging task since odor environments are typically turbulent. While time-averaged odor concentration varies smoothly with the distance to the source, instaneous concentrations are intermittent and obtaining stable averages takes longer than the typical intervals between animals’ navigation decisions. How to effectively sample from the odor distribution to determine sampling location is the focus in this article. To investigate which sampling strategies are most informative about the location of an odor source, we recorded three naturalistic stimuli with planar lased-induced fluorescence and used an information-theoretic approach to quantify the information that different sampling strategies provide about sampling location. Specifically, we compared multiple sampling strategies based on a fixed number of coding bits for encoding the olfactory stimulus. When the coding bits were all allocated to representing odor concentration at a single sensor, information rapidly saturated. Using the same number of coding bits in two sensors provides more information, as does coding multiple samples at different times. When accumulating multiple samples at a fixed location, the temporal sequence does not yield a large amount of information and can be averaged with minimal loss. Furthermore, we show that histogram-equalization is not the most efficient way to use coding bits when using the olfactory sample to determine location.

## Author summary

Navigating towards a food source or mating partner based on an animals’ sense of smell is a difficult task due to the complex spatiotemporal distribution of odor molecules. The most basic aspect of this task is the acquisition of samples from the environment. It is clear that odor concentration does not vary smoothly across space in many natural foraging environments. Using data from three different naturalistic environments, we compare different sampling strategies and assess their efficacy in determining the sources’ location. Our findings show that coarsely encoding the concentration of samples at separate sensors and/or multiple times provides more information than encoding fewer samples with higher resolution. Furthermore, coding resources should be focused on discriminating rare high-concentration odor samples, which are very informative about the sampling location. Such a nonlinear transformation can be implemented biologically by the receptor binding kinetics that bind odorants as a first stage of the sampling process. A further implication is that animals as well as computational models of algorithms can operate efficiently with a coarse representation of the odor concentration.

## Introduction

Diverse species throughout the animal kingdom use olfactory cues for navigation tasks critical to survival, including locating food sources and mating partners. However, olfactory navigation is not simple: odorants are often volatile and carried on rapidly changing currents, resulting in spatiotemporal distributions that are turbulent thereby defeating simple strategies such as gradient detection. Consequently, recent efforts at understanding olfactory navigation have focused on identifying the viable computational strategies for making navigation decisions [1, 2].

Here we focus on the most basic aspect of this process: how odor samples are encoded in the first place. Since sensory resources are finite, tradeoffs are inevitable. For example, resources may be allocated to encoding individual samples of odor concentration at a fine level of detail, or alternatively, to encoding multiple samples, either in space or in time, but at a coarser resolution for concentration. In this study, we investigate the implications of these and related tradeoffs, using the tools of information theory. Specifically, we compare an array of sampling and encoding strategies, asking to what extent they capture information about location within an olfactory environment.

There are several aspects of the statistics of an odor plume that can give clues as to the location of the source [3–7]. For example, the mean concentration varies smoothly in lateral and longitudinal directions. However, animals do not base their navigation decisions on mean concentration, as the time it takes to obtain stable estimates of mean concentration exceeds the typical time taken by animals to make navigation decisions [8–10]. Other olfactory features that have been proposed as useful for navigation decisions include the time between odor encounters [11–13] and intermittency (the probability of the odor concentration above threshold) [4]. However, as for mean concentration, obtaining stable estimates of these quantities takes more time than animals typically use for navigation decisions. Hence averaged quantities—even if aided by other sensory inputs—are probably not used to guide navigation decisions. These considerations motivate our focus on what can be learned from brief, localized samples. We do not address the issue of how to integrate odor samples with other sources of information.

A key starting point for our analysis is the explicit recognition that the resources available for sampling and encoding an odor environment are finite, and that it is natural to quantify these resources in terms of bits. This leads to the framework of information theory, which has the advantage that it minimizes the assumptions about the odor distribution.

As mentioned above, the sampling strategies we consider explore tradeoffs between the number of bits allocated to resolving concentration, and to sampling in space and time. The focus on these tradeoffs is motivated by the diversity of the sampling strategies that animals use. With regard to spatial aspects, most animals have two spatially separated antennae or nostrils which sample the olfactory environment, but the sensor spacing ranges from less than a mm to several cm. With regard to temporal aspects, insects’ olfactory receptors are continuously exposed to odorants, while rodents take periodic samples and adjust their sniff rate based on previous measurements [14–16].

In this article, we discuss sampling strategies based on local cues in light of how much information they provide about sampling location. To compare different sampling strategies, we computed the information that they conveyed about location, for three realistic olfactory environments. In each environment, odor concentration was empirically determined via physical measurements, planar laser-induced fluorescence [17]. We chose to use physical measurements of actual plumes not only to avoid the assumptions made by models of turbulence or the complexities of numerical simulations, but also because the non-idealities of physical measurements take into account the real-world issues that confront the olfactory navigator.

Although the three environments differed with regard to flow rate, turbulence, and proximity to a boundary, a number of commonalities emerged. First, precise measurement of odor concentration is generally not useful. That is, after allocating one or two bits to a coarse representation of odor concentration, more information about location is gained by using additional bits for encoding concentrations at nearby locations in space or time, than by using these bits to refine the representation of concentration. We also demonstrate that using “histogram equalization” as a strategy to discretize odor concentration—which is optimal to convey information about intensity per se [18]—is not optimal when the goal is to determine location. That is, the optimal strategy for low-level sensory encoding depends on the ultimate use of the information. Finally, with regard to sampling in time, we find that the additional information gained from multiple samples is preserved even if the temporal order of the samples is ignored, and this provides a rationale for simple post-receptoral processing strategies.

## Methods

### Plume measurements

Odor plume data were obtained experimentally using a surrogate odor (acetone) released in a turbulent flow within a benchtop low-speed wind tunnel. We imaged the odor structure using planar laser-induced fluorescence (PLIF); images were subsequently post-processed into calibrated matrices of normalized concentrations. We acquired three separate datasets varying in mean flow rates and proximity to a boundary.

The wind tunnel has a test section measuring 1 m long, by 0.3 m tall, by 0.3 m wide. We collected odor plume data at flow speeds of 5 cm/s and 10 cm/s. Ambient air enters the tunnel through a contraction section and passes through a turbulence grid consisting of 6.4 mm diameter rods with a 25.5 mm mesh spacing. Air exits the test section through a 15 cm long honeycomb section used to isolate the test section from a fan located in the downstream contraction. The odor surrogate was released isokinetically through a 9.5 mm diameter tube on the tunnel centerline. The tube orifice was located 10 cm downstream of the turbulence grid. For one dataset, named *boundary flow*, a false floor spanning the length and width of the test section was placed directly below the release tube.

Acetone vapor was used as a fluorescent odor surrogate. We generated the acetone vapor by bubbling a carrier gas through liquid acetone. Because acetone is denser than air, the carrier gas consisted of a mixture of air (59% v/v) and helium (41% v/v) such that the odor surrogate mixture was neutrally buoyant in the wind tunnel. We used a water bath to maintain the temperature of the odor mixture at ambient tunnel conditions.

A 1 mm thick light sheet from a Nd:YAG 266 nm pulsed laser illuminated the odor plume in the test section, causing acetone vapor in the odorant mixture to fluoresce with an intensity proportional to its concentration. The laser sheet enters and exits the tunnel through longitudinal slits along the sides of the test section. Plume fluorescence was imaged through a glass window in the tunnel using a high quantum efficiency sCMOS camera, with a bit depth of 16 bit, at a framerate of 15 Hz synchronized with the laser pulses. To enhance signal-to-noise, images were binned to (512x512) pixels corresponding to a spatial resolution of 0.74 mm/pixel. Raw images were processed to correct for background according to the equation

$$\begin{array}{c} c\left(t,x,y\right)=\frac{1}{a_{c}}\frac{I\left(t,x,y\right)}{F\left(x,y\right)}, \end{array}$$ (1)

where *c* is the normalized concentration, *I* is the image from the camera (with background signal subtracted) and *F* is the flatfield image (also with the background signal subtracted). The calibration coefficient, *a*_*c*_, was used to normalize the concentrations based on the source concentration at the tube exit.

Three datasets were collected, which had different combinations of wind tunnel flow rates and false floor configurations (Table 1). The first condition, named *fast flow*, had a mean free stream velocity of 10 cm/s, and the odor mixture was released into the center of the tunnel without a false floor. The second condition, named *slow flow*, had a free stream velocity of 5 cm/s, and acetone was also released into the center of the tunnel without a false floor. The third condition (*boundary flow*) had a free stream velocity of 10 cm/s, but in contrast to the first condition, acetone was released with the false floor in place. All datasets were collected in segments of 4 minutes. We had a total of 40 minutes (36000 frames) for the first and third condition, and 36 minutes (32400 frames) for the second dataset.

**Table 1:** Overview of different datasets.

dataset	label	*v*_avg._ [cm/s]	region [cm] (pixels)	boundary
*fast flow*	A	10	30 × 16 (406 × 216)	no
*slow flow*	B	5	30 × 16 (406 × 216)	no
*boundary flow*	C	10	30 × 16 (406 × 216)	yes

**Fig 1:**
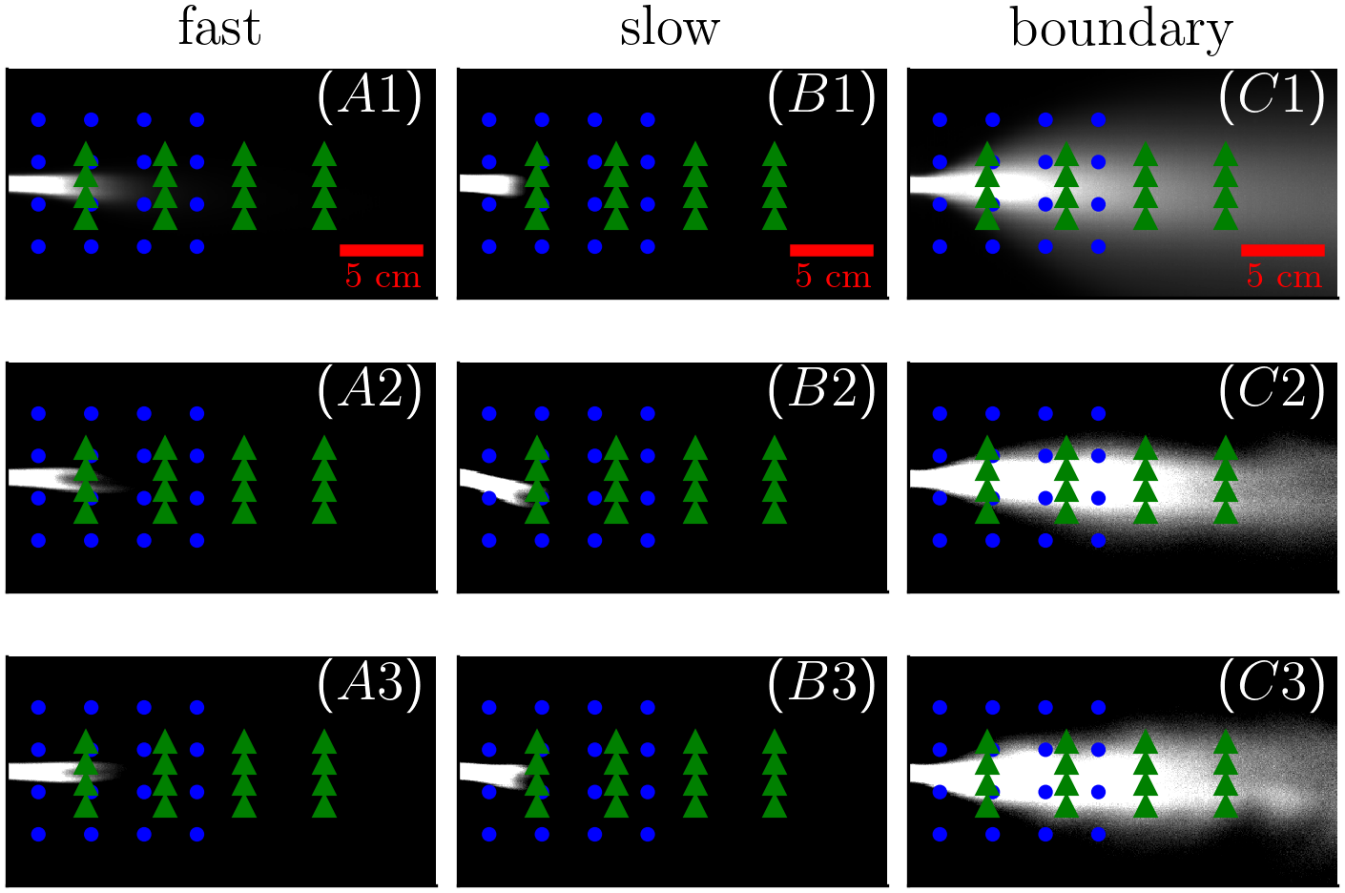
Snapshots of plume measurements. Three different flow conditions were measured (*A*1 − *A*3 *fast flow*, *B*1 − *B*3 *slow flow*, and *C*1 − *C*3 *boundary flow*, for details see Table 1). Top row shows the time-averaged odor concentration for each of the conditions. All concentrations are shown relative to the source concentration. The green triangles and blue dots correspond to the two 16-location grids. A scalebar indicating 5 cm is shown at bottom right corner of *A*1-*C*1. The middle and bottom rows (*A*2-*C*2, *A*3-*C*3) show two typical snapshots of the instantaneous odor concentration for each of the flow conditions.

The matrices of normalized concentrations provide a natural coordinate system. Time-averaged odor concentrations and two typical snapshots for the three conditions are shown in Fig 1. To compare olfactory cues across different flow conditions, we chose two grids of 16 locations in each olfactory landscape ($G_{\text{narrow}}$ and $G_{\text{wide}}$). Coordinates of the locations for the grid choices (inlet location at the origin) are:

$$\begin{array}{c} \begin{array}{cc} G_{\text{narrow}} & =\{(x,y)\;|\;x=(2.2,5.9,9.6,13.3)\;\text{cm},\;y=(-4.4,-1.5,1.5,4.4)\;\text{cm}\},\\ G_{\text{wide}} & =\{(x,y)\;|\;x=(5.6,11.1,16.7,22.2)\;\text{cm},\;y=(-2.6,-1.1,1.1,2.6)\;\text{cm}\}. \end{array} \end{array}$$ (2)

The two grids were chosen to capture the environment close to the source and further away from it above and below the centerline. The locations are indicated as blue circles ($G_{\text{narrow}}$) and green triangles ($G_{\text{wide}}$) in Fig 1. The distances between gridpoints and the odor source are directly relevant to walking flies and other small insects.

Probability distributions of the odor concentrations of the upper half of all grid points are shown in S1 Fig.

### Mutual information

Our primary goal is to quantify the extent to which a small number of samples of odor concentration within a plume provide information about the location of the sample. A principled approach is to use Shannon’s mutual information (MI) [19] for this purpose. That is, using entropy as a measure of uncertainty, we will determine the extent to which a given encoding scheme reduces the uncertainty about the location of the sample. Thus, our two variables of interest are location (L) and discretized odor samples (M); these are related in a complex statistical fashion. Specifically, this analysis quantifies the ability to discriminate between the 16 locations of either $G_{\text{narrow}}$ or $G_{\text{wide}}$ when the only available information comes from odor intensity samples.

The choice of 16 locations per grid is somewhat arbitrary, however, in order to get stable information estimates with a given amount of data one trades off the number of locations with the number of bits using for odor coding. We settled on 16 locations as they capture a good proportion of the environment while allowing for the analysis of coding of odor samples with up to 10 bits.

As is well-known, the MI between two random variables L and M is [19, 20]:

$$\begin{array}{c} I\left(L,M\right)=H\left(L\right)-\sum_{m\in M}p\left(m\right)H\left(L|m\right), \end{array}$$ (3)

where H(L) is the (unconditional) entropy of L, and H(L|m) is the entropy of the distribution of L conditional on m∈M.

In our context, L is the set of sampling locations $G_{\text{narrow}}$ or $G_{\text{wide}}$ and m∈M is a measurement of the normalized odor concentration *c*(*t*, *x*, *y*). The specific representation of *c* as a (coarser) measurement *m* is an integral part of the encoding schemes we consider.

We assume that the a priori probability of the locations l∈L are equal. It follows that the unconditional entropy is

$$\begin{array}{c} H\left(L\right)=-\sum_{l\in L}p\left(l\right)\log_{2}\;p\left(l\right)=\log\left(|L|\right), \end{array}$$ (4)

where |L| is the number of sampling locations. Note that the MI (Eq 3) is a property of the grid as a whole, not the individual points. Since all |L| grid points have the same a priori probability, the upper bound of the MI is $\log_{2}\left(|L|\right)$. If the navigator has $\log_{2}\left(|L|\right)$ bits of information then it knows its location on the grid unambigously.

Posterior (conditional) distributions *p*(*l*|*m*) were calculated by Bayes theorem. Specifically, we binned the odor concentrations *c* at each location *p*(*m*|*l*) and then normalized the likelihoods by *p*(*m*). The entropy of these conditional distributions are given by

$$\begin{array}{c} H\left(L|m\right)=-\sum_{l\in L}p\left(l|m\right)\log_{2}p\left(l|m\right). \end{array}$$ (5)

This quantity, weighted by the probability that sample *m* occurs p(*m*), is summed over all m∈M to determine the average conditional entropy in Eq (3).

We used two contrasting strategies for representing the odor concentration as discrete symbols (bins). In the first strategy, we divided the data into equal quantiles, *i.e.* we chose boundaries such that the distribution *p*(*m*) is uniform. This histogram-equalization procedure maximizes the information conveyed about the odor concentration (*i.e.*, M) [20, chap.2], but does not necessarily maximize the information conveyed about sampling location. In the second strategy, we adjusted these bin boundaries to increase the amount of information about location. Because finding the bin boundaries that yield an absolute maximum is a multidimensional discrete optimization problem, we used the following “greedy” iterative strategy to find an approximate maximum. The first bin boundary is chosen to maximize I(L,M), and is identified by an exhaustive search of the range of concentrations. Then, iteratively, the *k*-th boundary is chosen to maximize I(L,M) while keeping the *k* − 1 bin boundaries fixed. This is also a one-dimensional search over the range of concentrations, and leads to a binary subdivision of one of the bins determined at the previous step. For analyses in which the odor at multiple temporal or spatial samples is encoded, we used the bin boundaries determined from these single-sample optimizations.

The encoding strategies we considered are specified not only by the way that each sample is encoded (i.e., the bin boundaries), but also by the number of spatial samples *r*_spat_ and the number of temporal samples *r*_temp_. Specifically,

$$\begin{array}{c} S(n_{\text{bits}};r_{\text{spat}},r_{\text{temp}}), \end{array}$$ (6)

denotes an encoding strategy that uses *n*_bits_ to discretize odor intensity, applies this discretization to *r*_spat_ samples at nearby locations obtained at *r*_temp_ points in time. Note that the number of bins used to discretize odor concentration is given by $2^{n_{\text{bits}}}$. When investigating strategies with two sensors (*r*_spat_ = 2), we take two samples at a distance of 0.3 cm (four pixels) centered around the locations specified in Eq (2).

For sampling strategies specified by the notation of Eq (6), bin boundaries are obtained by histogram equalization. To indicate that the “greedy” strategy has been used for obtaining bin boundaries, we use the symbol $n_{\text{bits}}^{*}$. The total number of bits used for encoding a sample *m* is given by *n*_bits_ ⋅ *r*_spat_ ⋅ *r*_temp_ (or $n_{\text{bits}}^{*}\cdot r_{\text{spat}}\cdot r_{\text{temp}}$).

To ensure that our results do not reflect the idiosyncrasies of odor concentrations at specific locations, all calculations were repeated after jittering the grid location. Specifically, the grid was rigidly moved from its standard location (as given in Eq (2)) by 0.74–2.22 mm (1-3 pixels) in *x* and *y* directions, yielding a total of 49 placements. In all figures of the results section, mutual information at these jittered locations are shown as shaded blue and green regions.

#### Bias in the information estimates

As described above, we used the “plug-in” estimator for entropy since this makes no assumptions about the nature of the distributions. However this estimator (as well as any other entropy estimator) is subject to bias due to finite sample size [21, 22]. Since fewer samples are available for estimating posterior distributions *p*(*m*|*l*) compared to *p*(*m*), H(L|M) is more biased than H(L), and the estimate of mutual information I(L,M) is therefore upwardly biased. This consideration, along with the need to keep the bias small, limited the range of coding schemes that we considered.

To demonstrate that the bias was indeed small for the coding schemes considered, we assessed it via the method of [23, 24]. Here, mutual information is expanded as a series in 1/*N*, where *N* is the number of samples. Within the range of validity of the expansion, the 1/*N*-term of this series is the bias estimate. S6 Fig in the Supporting Information section demonstrates the validity of the asymptotic expansion for some coding schemes used in our analyses (by estimating information from smaller subsets of the full dataset). The bias-corrected information is the intercept with the ordinate. Given that the slope of the 1/*N*-term is virtually identical for the jittered grid locations, we computed similar asymptotic expansions for the centered locations of the narrow- and *wide grid* of all coding schemes and subtracted the bias estimate from all mutual information curves that involve more than one bit.

## Results

**Fig 2:**
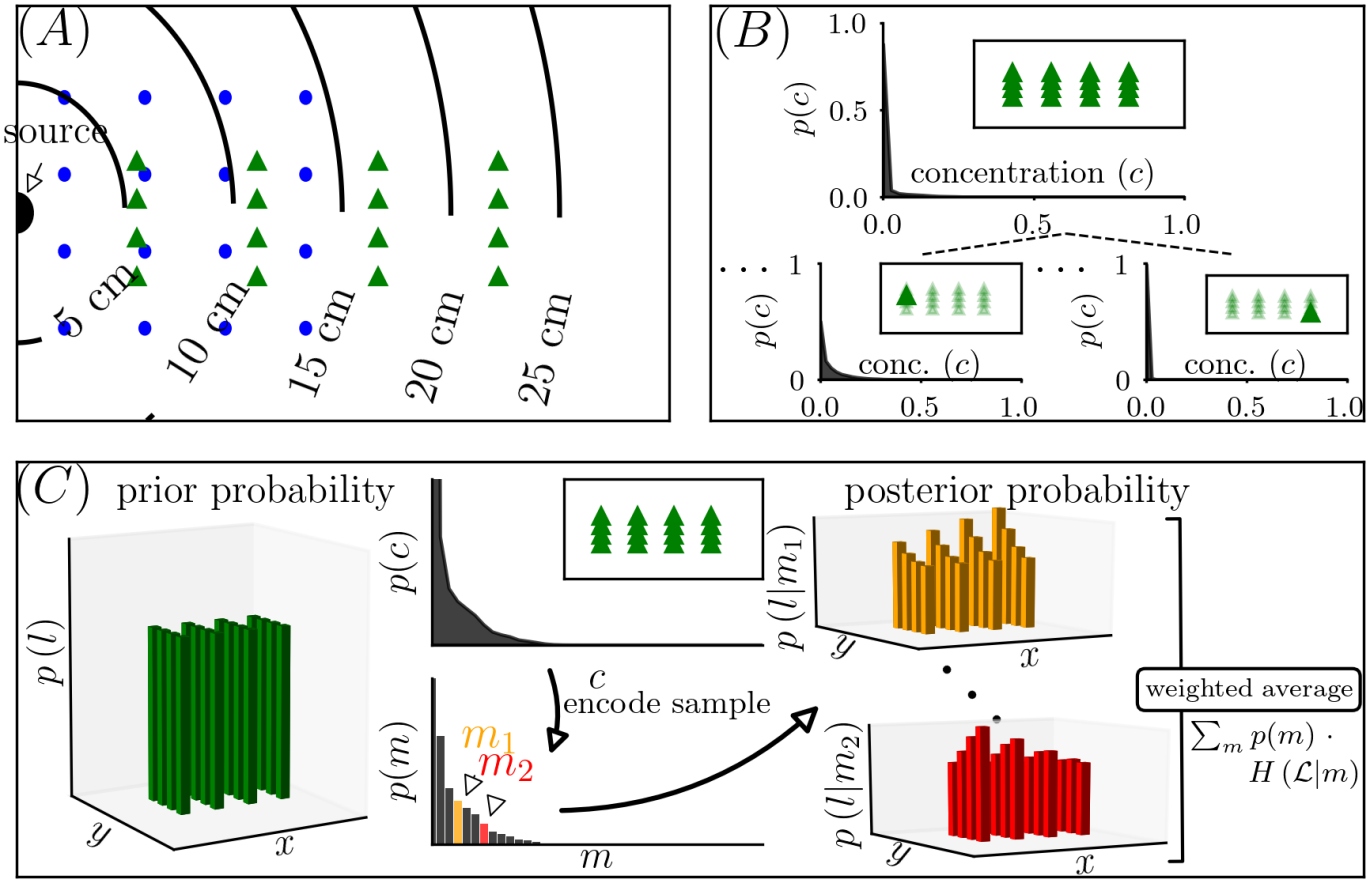
Overview of the analysis. (*A*) shows the distance to the odor source for each of the locations of the two 16-location grid choices (green triangles and blue dots) and the imaged area. (*B*) shows probability distributions of the normalized odor concentrations (normalized by source concentration). The top panel shows the composite distribution, using data of all 16 locations, for the *wide grid* in the *fast flow* condition. Below are probability distributions for two selected locations (as indicated by solid green triangles in the inset). (*C*) Schematic of the information-theoretic analysis. On the left is the prior probability, which is equal for all 16 locations (depicted by equal height bars). The second column shows the composite distribution (top) and the result of discretizing it into *M* levels (bottom). The discretization recognizes that the observer has limited coding resources available. A sample from the discretized distribution results in an updated belief of where the observer is, represented by the posterior distribution. Two example posterior distributions are shown, one for drawing the sample *m*_1_ (orange), and one for drawing the sample *m*_2_ (red). Posterior distributions have lower entropy than the prior distribution, since the locations are no longer equally likely. The difference of the entropy of the prior and the weighted average of the posterior entropy is the information a navigator can expect to learn about location with a given sampling strategy. Note that this panel is a diagram for the analysis of encoding a single sample at a single time ($S(n_{\text{bits}};1,1)$); an analogous strategy is used to analyze encoding with two sensors and/or encoding of multiple samples in time ($S(n_{\text{bits}};r_{\text{spat}},r_{\text{temp}}$)).

A schematic overview of our analysis can be seen in Fig 2. We chose two grids of 16 locations for independent analyses of estimating information that sampling from the odor field provides about the navigator’s sampling location. Distance to the odor source is indicated in panel (*A*). Practical considerations restrict the experimental analysis to distances in the cm range. These are directly relevant to small insects.

Each of the 16 locations has a different distribution of odor probabilities as diagrammed in Fig 2*B*. These were determined experimentally by PLIF, as described in the methods section.

The approach of evaluating a sampling strategy based on the amount of information it provides about location is cartooned in Fig 2*C*. A navigator starts with no knowledge of its location, and hence assigns an equal probability to be in any of the 16 grid locations (L). The navigator samples the environment and computes a posterior distribution. Based on the odor sample, the posterior distribution weights the locations unequally. It therefore has a lower entropy than the prior distribution. The average reduction in entropy is, by definition, the MI, and this quantifies the partial knowledge that an odor sample conveys about location.

The main theme of this analysis is that an observer does not have access to the raw concentration, but only to a degraded version of it. In Fig 2, we diagram the scenario in which the observer discretizes a single odor sample into a specific number of levels; this discretized version of the odor, rather than the raw odor concentration itself, is used to compute the posterior distribution. As described below, we compare the utility of this sampling scheme to schemes in which several samples, in time or in space, are encoded.

In keeping with the laboratory setting, we describe the analysis in terms of a fixed odor source and an unknown location. Since the relevant quantity is the displacement between the navigator and the source, this formulation corresponds to an actual navigation task, in which the navigator knows its location and attempts to infer the location of the source.

### Three ways to allocate coding resources

We considered encoding schemes that probed the three basic ways in which resources could be allocated to encoding the odor measurements: for resolving concentration, for sampling across space, and for sampling across time.

Here and in the other analyses below, parallel calculations were carried out for three odor environments: *fast flow (A), slow flow (B)* and *boundary flow (C)*, and for two sets of locations (*narrow grid* (blue) and *wide grid* (green)) within each environment. The *fast flow* and *boundary flow* conditions have the fastest inlet flow of 10 cm/s, but the *boundary flow* dataset was taken near a boundary where the odor surrogate’s dynamics are affected by boundary layer effects. Hence, *boundary flow* is the condition were diffusion has the biggest impact; see Methods for details. As a consequence of the more diffusive regime of the *boundary flow* condition the mutual information values we obtained for this condition are somewhat higher than in the other two conditions. The *slow flow* dataset has an inlet velocity of 5 cm/s. Except as noted, the analyses with different datasets and different grid choices yielded similar results.

**Fig 3:**
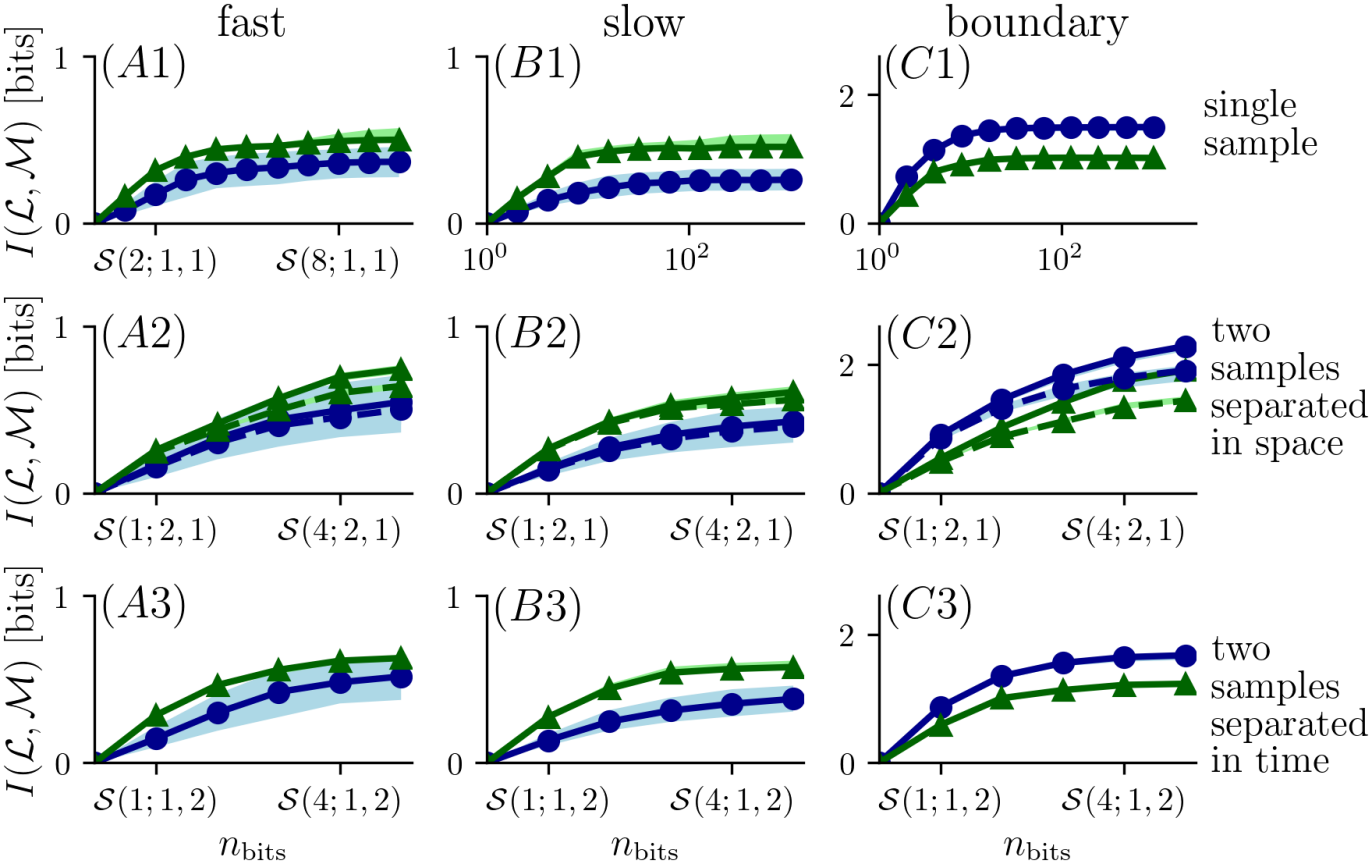
Mutual information between location (L) and measurement (M) for different encoding strategies. The figure indices *A*, *B* and *C* correspond to the different flow conditions (*fast flow*, *slow flow* and *boundary flow*); the rows 1-3 compare different encoding strategies. In the first row the mutual information is calculated based on single samples taken at either the *narrow grid* (blue curves correspond to blue circles in Fig 1) or the *wide grid* (green curves correspond to green triangles in Fig 1). *A*2-*C*2: Coding schemes with increasing number of bits assigned to two sensors. Two samples separated in space were taken at a single time. Solid lines show information using knowledge of which sample occurs in which sensor; dashed line shows information ignoring which of two sensors measures which sample. *A*3-*C*3: Assigning bits to two temporal samples taken at the same location with a delay of 1.6 s. In all panels, bold curves correspond to estimates for the locations as given in Eq (2) and shaded regions correspond to information estimates for jittered locations (see Methods).

Fig 3*A*1–3*C*1 shows results for strategies that devote all bits to encoding concentration at one point in space and time ($S(n_{\text{bits}};1,1)$). As the resolution for odor concentration increases, so does MI, but only up to a point: once four bits are used to resolving odor concentration, additional resolution yields only minimal increases in MI.

When measurements are made at two sensor locations (transversely separated by 0.3 cm), using additional bits for coding allows MI to increase beyond the plateau encountered with a single sensor (Fig 3*A*2–3*C*2). The benefit of spatial sampling is not merely the result of having two independent samples. Specifically, MI computed after ignoring which sample corresponded to which sensor was smaller, by up to 0.1 to 0.2 bits (dashed curves in Fig 3*A*2–3*C*2), than the MI conveyed by a coding scheme that keeps track of which sample is which. This indicates that sampling with two sensors enables extraction of a spatial feature of the odor plume that varies along the vertical axis. This trend is also true for different spacing between two sensors, as shown for half intersensor distance and double intersensor distance in S5 Fig. Note that in the *boundary flow* condition, the curves continue to increase rapidly at the limits of measurement, suggesting that MI is not close to saturation.

Encoding odor measurements at two consecutive times (separated by 1.6 s) also increases MI beyond the plateau of a single sample, but not by as much as for two spatial samples (Fig 3*A*3–3*C*3). While each additional bit used for resolving the concentration of two consecutive samples provides greater MI, the increases become progressively less, suggesting that MI has reached a plateau when five bits of resolution are devoted to two samples separated in time. Virtually identical results are obtained for longer intervals between samples; this is expected since MI reaches an asymptotic value as a function of sampling interval (see section *temporal encoding strategies* below).

In the above analysis, we discretized the odor concentration into sub-intervals of equal probability, as this histogram-equalization procedure provides the greatest amount of information about the odor concentration itself [18, 20]. However, this does not yield the maximal MI about location, so we carried out a further analysis that explored the discretization strategy.

**Fig 4:**
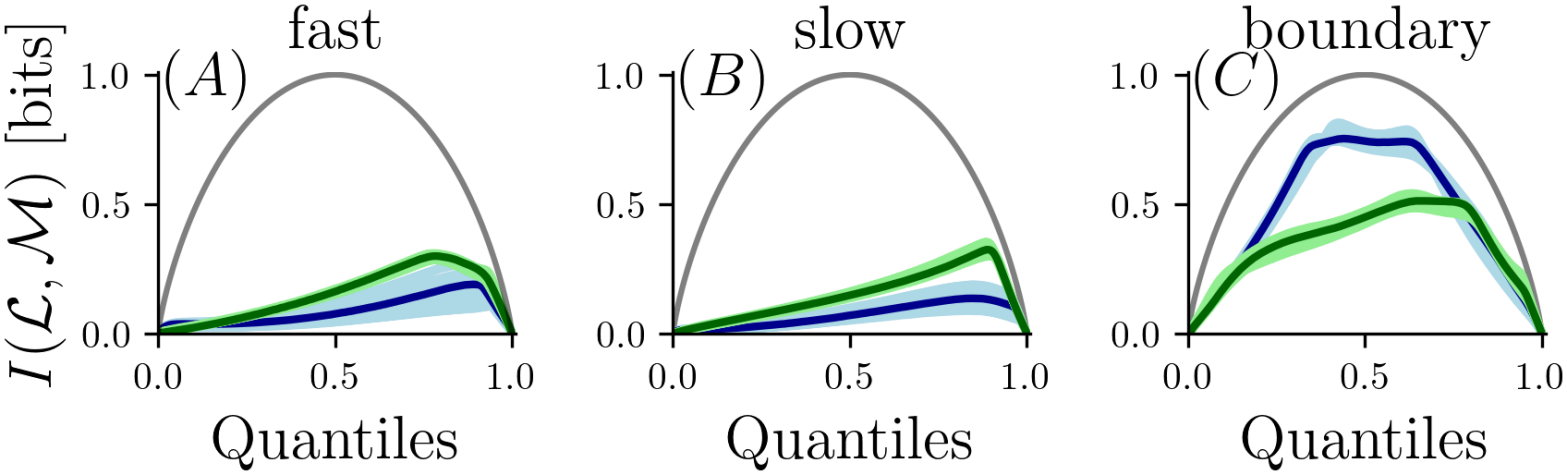
Mutual information based on binarization of single odor measurements parametric in the threshold. If all concentrations would be equally likely, chosing the median would provide most information about concentration (indicated as gray curves for comparison). In condition *A* (*fast flow*) and *B* (*slow flow*) chosing a threshold above the median increases the amount of information about location. The information curve is flat for condition *C* (*boundary flow*). Color coding as in Fig 3.

For the simple case of discretization into two levels, we show how the MI depends on the binarization threshold in Fig 4. For the *boundary flow* condition (*C*) the information curves are flat over a large range for the *narrow grid*, and has a maximum above the median for the *wide grid*. For the *fast flow* (*A*) and *slow flow* (*B*) condition the maximum of information is obtained when the threshold is above the median for both grids. This suggests the most informative samples about location occur at high concentration. A threshold above the median exploits this feature of the odor statistics and allows better discriminability between locations. A comparison between the bin boundaries obtained by histogram-equalization and the optimal bin boundary when binarizing odor can be seen in S2 Fig. It is evident that the optimal bin boundary occurs at a higher concentration than the median for all but the *narrow grid* of the most diffusive condition.

To investigate how a different choice of bin boundaries affects the results of Fig 3, we implemented a “greedy” partitioning scheme (see Methods) in which the first cutpoint was chosen to yield the maximal MI about location, and then successive cutpoints were chosen so that each maximized the MI about location, given the previous partitioning. Results (see S3 Fig) were very similar to the above analysis based on histogram-equalized bins (Fig 3). Although one- and two-bit encoding schemes (two to four partitions) yielded more MI than histogram equalization, the plateau seen in row 1 of Fig 3 was essentially unchanged. The advantage of encoding schemes based on two spatial or two temporal samples persisted.

### Comparing different encoding strategy based on two sensors

The above findings show that overall, there is surprisingly little benefit to allocating coding bits to resolving odor concentration, compared to allocating them to capture several samples across space or time. We hypothesized that resolution of odor concentration might become more important in regimes that were more diffusive, especially when coupled with sampling at two locations. To investigate this hypothesis, we compared coding schemes in which the same number of bits (four bits at each of two spatial samples) were allocated to one, two, or four samples in time, and in which the spatial sampling was across the flow axis (as in Fig 3), or along the flow axis.

**Fig 5:**
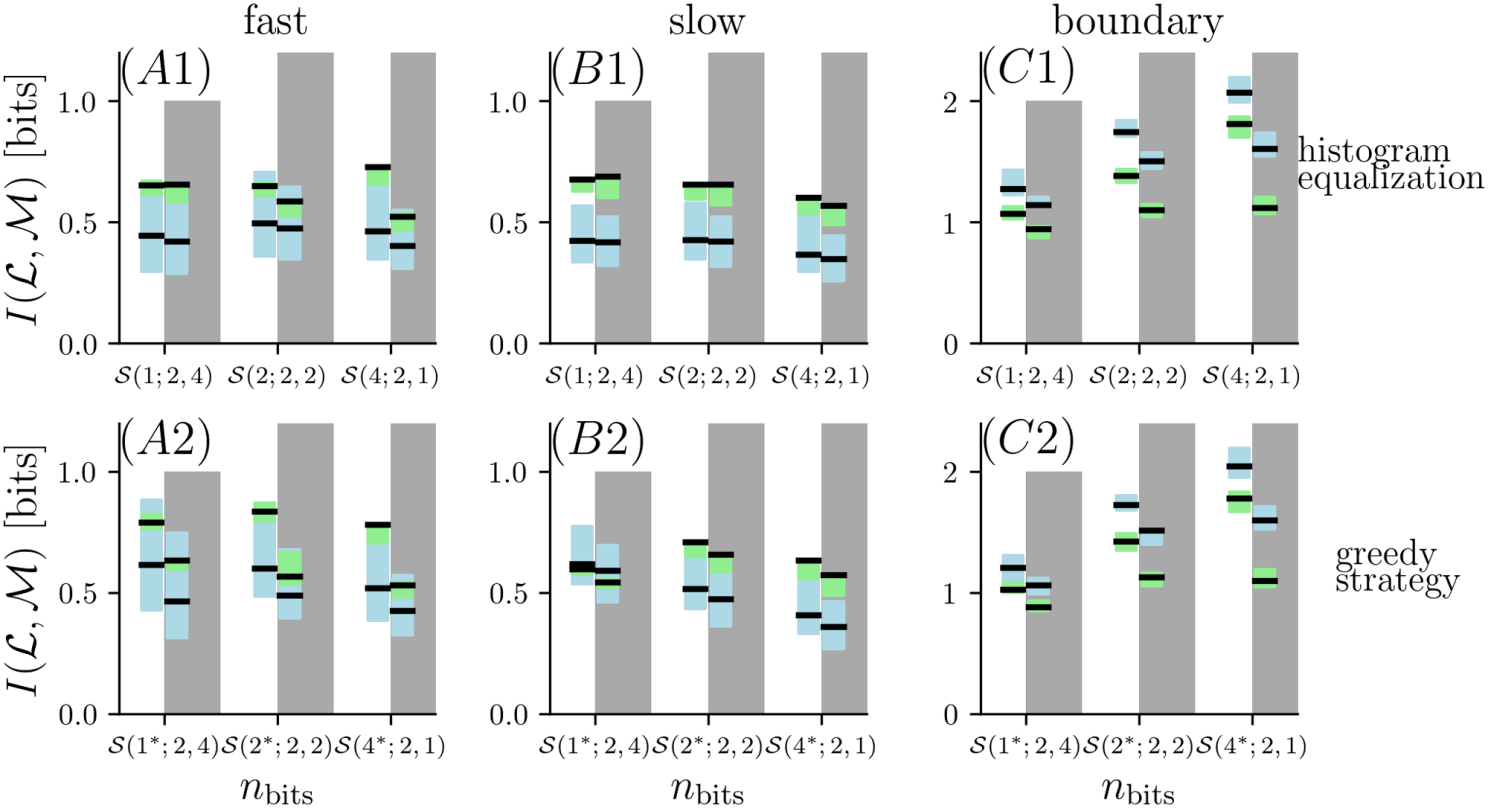
Mutual information for different encoding strategies based on two sensors. Eight bits are assigned in three different strategies to two sensors. The blue shaded region marks the mutual information for the narrow grid and the green shaded region marks the mutual information for the wide grid (black lines for centered locations, shaded regions for jittered locations). For each strategy, the left columns (white region) correspond to a sensor alignment transversal to the centerline of the imaged area. The right columns (gray shaded region) correspond to a sensor alignment longitudinal to the center line. The first row (*A*1—*C*1) corresponds to chosing bins boundaries with histogram equalization, the second row (*A*2—*C*2) corresponds to chosing bin boundaries according to the “greedy” method as explained in the text.

Fig 5 shows that this hypothesis is supported. Considering first bin boundaries based on histogram equalization, and sensor locations across the flow axis (unshaded portions of plots in first row of Fig 5), two or more bits were only beneficial for the most diffusive environment *boundary flow* (Fig 5*C*). Likewise, for sensor locations along the flow axis (shaded half of each subplot), more than one bit of resolution was only helpful in this environment (*boundary flow* (Fig 5*C*)).

Similar conclusions are reached when bin boundaries are determined via the “greedy” binning procedure: more than one bit of resolution for odor concentration is only useful in the most diffusive environment (*boundary flow* (Fig 5*C*)), and has the greatest benefit when the two sensors are across to the axis of flow. In the *fast flow* condition, increasing resolution while decreasing the number of samples in time makes little difference (Fig 5*A*), and for the *slow flow* condition (Fig 5*B*), increasing resolution while decreasing the number of samples leads to a loss of information about location for either sensor orientation.

In sum, the results of Figs 4*C*, 5*C*1 and 5*C*2 show that in a diffusive regime the exact choice of bin boundaries is not important, but devoting up to four bits to concentration resolution has a benefit over accumulating multiple temporal samples. When the flow conditions are more turbulent, a navigator benefits from classifying multiple odor samples at coarser resolution (Fig 5*A* and 5*B*), but the choice of the discretization threshold becomes important (Fig 4*A* and 4*B*). Consistent across conditions, sampling across the odor plume yielded more MI than sampling along the mean flow direction (white vs. gray shaded regions in Fig 5).

### Temporal encoding strategies

#### Optimal time interval between samples

The utility of multiple samples at sequential times is likely to depend on how the sampling interval interacts with flow conditions: for intervals at which odor concentrations are strongly correlated, multiple samples are not likely to provide a substantial increase in MI.

**Fig 6:**
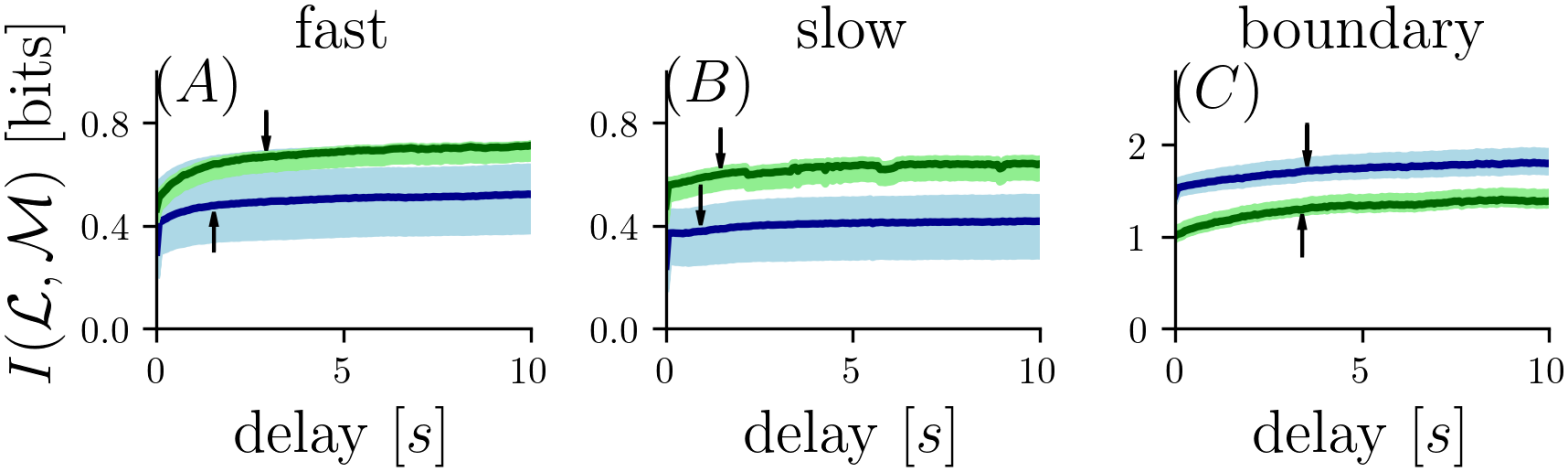
Comparison of mutual information of two consecutive samples taken at the same location (S(5;1,2)) parametric in the time interval between the two samples. Arrow indicates time at which the second sample provides 80% of the asymptotic value. Color code as in Fig 3.

This interdependence is investigated in Fig 6, which shows the MI for two samples obtained across a range of time separations. As in the last data point of Fig 3*A*3–3*C*3, all datasets have five bits assigned to each of two samples.

The arrow in Fig 6 indicates the time at which a single sample provides 80% of the asymptotic value of information (*τ*_80_) of two samples. Relatively short values are seen for the *narrow grid* of the *fast flow* dataset (*τ*_80_ ≈ 1.6 s blue curves in Fig 6*A*) and the *slow flow* dataset for both grids (*τ*_80_ ≈ 1 s *narrow grid*, blue curves and *τ*_80_ ≈ 1.5 s *wide grid*, green curves in Fig 6*B*).

For the *boundary flow* dataset *τ*_80_ is approximately 3.5 s (Fig 6*B*) for both grids. Thus, MI rises more quickly in the conditions *fast flow* (Fig 6*A*) and *slow flow* (Fig 6*B*) compared with the *boundary flow* condition. This finding is unsurprising, since diffusion has the largest impact in the *boundary flow* conditions and likely accounts for the larger value of *τ*_80_. However, the benefit of increasing the inter-sample interval reaches an asymptote in all cases, as would be expected once the interval is sufficiently long so that the samples are independent.

#### Information in the temporal sequence of measurements

To focus on the interaction of concentration resolution and number of temporal samples, we compared strategies that sampled at a single location, and traded off the number of bits allocated to resolving concentration at each sample, with the number of samples. In each case, a total of ten bits were used.

**Fig 7:**
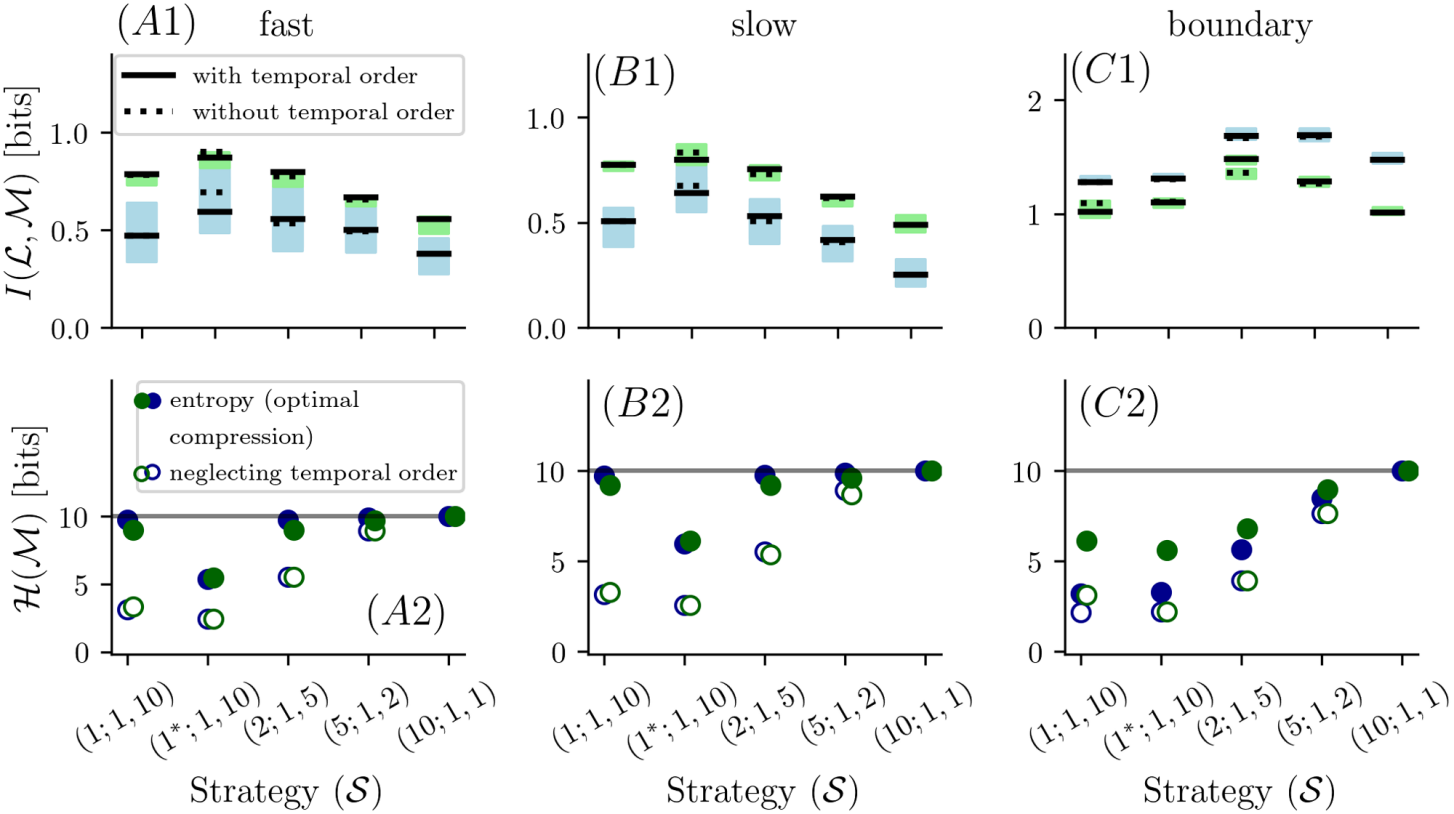
Top row shows the mutual information for different strategies of assigning ten bits into multiple consecutive samples for single sensors. The black solid lines correspond to mutual information with knowledge of the temporal sequence in which samples occur. The dashed black lines correspond to mutual information where the temporal sequence is ignored. The blue and green shaded regions correspond to information for the jittered locations of the narrow- and *wide grid*. Second row (*A*2-*C*2) shows the entropy for the corresponding strategies. The gray lines indicate the entropy of the incompressible odor measurements (S(10;1,1)). Full blue and green circles represent the entropy of the measurements at the narrow and *wide grid* locations. Hollow circles correspond to the measured sequence where the temporal sequence is ignored.

When using histogram-equalization, for almost all flow environments and grid choices, devoting all bits to single measurements provides the lowest amount of information (see Fig 7*A*1–7*C*1), and the most informative strategy is to assign two bits to concentration resolution for five temporal samples (S(2;1,5)). However, for the *fast flow* and *slow flow* environments, one bit of resolution provided even more information, provided that the threshold was chosen in the optimal way ($S(1^{*};1,10)$).

Although encoding multiple samples provides greater information than a single sample, keeping track of the specific sequence of the samples (*i.e.* their temporal order) carries relatively little information. This is shown by the difference between the solid black lines and the dashed lines in Fig 7*A*1–7*C*1. (For the optimized threshold measurements of MI in Fig 7*A*1 and 7*B*1 the MI seems larger when ignoring the temporal order; this apparent anomaly is a consequence of data limitations and debiasing, since the bias on MI estimates that make use of temporal order is higher than bias of MI estimates that ignore temporal order).

#### Compression of odor measurements

Until now, we compared encoding strategies based on the number of bits required for a “naïve” implementation, in which *n*_bits_*r*_spat_*r*_temp_ bits are used to represent each word of the code $S(n_{\text{bits}};r_{\text{spat}},r_{\text{temp}})$. However, these naïve representations are typically compressible, since the words do not occur with equal frequency. Specifically, the entropy of the distribution of code words provides an estimate of the extent to which it may be compressed without loss [19], [20, chap.5]. Further compression may be possible if correlations in the sequence of code words are present, but we ignore any such correlations here.

These distribution entropies are shown by the filled symbols in Fig 7*A*2–7*C*2 for the codes of Fig 7*A*1–7*C*1. As expected, when bin boundaries are chosen by histogram equalization and there is only one temporal sample S(10;1,1), all code words are equally likely and entropy is *n*_bits_. However, when a single code word encompasses two temporal or more temporal samples, the words are unequally distributed, and lossless compression is possible. The amount of lossless compression is strongest for the codes with optimized binarization levels ($S(1^{*};1,10)$).

Since temporal order of the samples that constitute a code word contributed only a modest amount of information (solid vs. dotted lines in Fig 7 top row), we also considered the extent to which ignoring temporal order would allow for further compression. As shown by the hollow circles in Fig 7, this enables approximately a factor of two of further compression, quite substantial compared to the minimal amount of information lost when temporal order is neglected.

## Discussion

In olfactory navigation, one of the main challenges is the complexity of the typical odor environment. Typical environments are turbulent, and are characterized by short bursts of high odor intensity interspersed with long durations of low odor intensity [7]. Thus, simple strategies based on the gradient are likely to fail, and it is not obvious which aspects of the environment—as sampled locally by a navigating organism—are most useful in determining location. To address this question, without making specific assumptions about the form of these statistics or the navigation strategy per se, we used an information-theoretic approach: we compared different strategies for encoding odor samples in terms of the information they carry about location. This information-theoretic approach is similar in spirit to a study investigating the feasibility of communication via modulated release of pheromones in idealized environments [25].

Specifically, we examined encoding schemes with a fixed amount of coding resources (bits), and evaluated codes that allocated these bits to encoding odor concentration in a coarse vs. fine manner, or at one vs. two locations, or at one vs. multiple times. In the three odor environments we considered, there was little benefit in resolving odor concentration with high accuracy for single samples. The range where additional bits stop improving the information significantly depends somewhat on the binning strategy. If the bins are allocated according to histogram equalization, information plateaus when 3 or 4 bits per sample are allocated to concentration. But with a “greedy” binning procedure, this plateau is reached sooner.

Interestingly, a “greedy” binning strategy is effective in determining location even when only using one or two bits to resolve odor concentration (binarizing or dividing odor concentrations into four levels). Merely binarizing the odor concentration—*i.e.*, encoding odor concentration as either “low” or “high”—reveals more than half of the maximal information in all conditions but the least turbulent. The binarizing cutpoint that maximizes information about location is higher than the cutpoint that maximizes information about odor itself, *i.e.*, the median. For the more turbulent regimes, setting the cutpoint at the optimal level for location yields almost double the amount of information than would be yielded by a median cutpoint. The potential advantages of a “greedy” binning strategy over histogram equalization are even greater when one considers that for greedy strategies, the resulting distribution of encoded measurements has lower entropy than for histogram equalization, and thus, is amenable to simple non-lossy compression.

Sampling odor at two locations, or several times, breaks through the plateau that is reached as further bits are allocated to odor resolution. These strategies are always more informative than devoting all bits to encoding concentration at a single location when more than four bits are available. In the three environments we examined, a second sample separated in space carries more information than a second sample separated in time. A considerable amount of information is gained by comparing which sensor registers which sample. The amount of this increase depends on the sensor spacing, with larger spacings yielding a larger increase in information (see S5 Fig).

Comparison of concentrations in two sensors is advantageous in both diffusive and turbulent environments. The advantage is to be expected in a diffusive environment, since this comparison yields an estimate of the gradient, but interestingly, our findings show that it persists in turbulent environments as well.

Allocating the same number of bits to multiple temporal samples also increases the amount of information transmitted about location. Consistent across odor environments, the sequence of samples, per se, matters very little. In contrast to the benefit of keeping track of which spatial sample is which, we find little utility in tracking the specific sequence of temporal samples. In other words, ignoring the sequence of measurements across time is a form of lossy compression that results in only a minimal loss of information about location. The effectiveness of this compression (i.e., the ratio of the information about location to the output entropy) is greater for a greedy binning strategy than for histogram-equalization.

### Implications for odor coding systems

We now discuss the implications of our findings, first with regard to sensation and then with regard to navigation algorithms. As a starting point, we consider the simple scenario of a sensory system confronted with a continuous and widely varying input, but limited in the number of symbols that it can use for encoding. As is well-known, information is maximized when each of the symbols is used equally often, i.e., histogram equalization. Histogram equalization can be implemented as a nonlinearity applied to the input prior to producing a neural output [18]. For a positively skewed distribution, such as light intensities or odor intensities, the nonlinearity is a highly compressive one, so that it takes into account the rarity of very large inputs.

Here, however, we consider the task of maximizing information not about the sensory signal itself, but about location—which is related to odor concentration in a complex, stochastic manner. As we showed, most of the available information about location can be conveyed by a coarse discretization of the sensory range—in fact, by binarization. However, this only holds if the cutpoint is properly chosen. In the two more turbulent odor environments considered here, the optimal cutpoint is substantially higher than the median, which is the cutpoint associated with histogram equalization (see Fig 4). That is, discriminations in the upper range of odor concentrations play a disproportionately greater role in determining location, than in reconstructing the input per se. Correspondingly, implementation of this encoding requires a nonlinearity that is less compressive for higher intensities than histogram equalization.

Optimal adaptation strategies, in the sense of being maximally informative, under naturalistic stimuli are (to our knowledge) unknown. The problem of optimally discretizing a signal is not just an olfactory problem but applies to other sensory modalities which face resource constraints as well (*e.g.* vision [26–28]).

While it is difficult to imagine a biologically-plausible mechanism that achieves the precisely optimal nonlinearity for conveying information about location, there is a simple and plausible mechanism that can achieve an approximation: ligand-receptor binding in olfactory receptor neurons [29]. In steady-state, this mechanism generates a nonlinear encoding described by the Hill equation [30]. This transformation compresses signals at high concentrations, because receptors become occupied, and more ligand is required to activate the remaining receptors [31]. Thus, the degree of compression depends on the apparent dissocation constant *K*_*d*_, the odorant concentration at which half of the receptors are occupied. Setting *K*_*d*_ at the median odor concentration corresponds to histogram equalization: half of the time the ligand binding will be below the median, and half of the time it will be above.

Interestingly, setting *K*_*d*_ at the mean concentration, rather than the median, leads to less compression than histogram equalization. This is because the measured odor concentrations are positively skewed. Since the mean odor concentration is larger than the median, this setting will produce a response that is less than half-maximal most of the time. Such a coding strategy results in more information about location than histogram equalization, as we have outlined above (see Fig 4). In order to implement this strategy, olfactory receptors or receptor neurons would have to have an apparent *K*_*d*_ close to the mean concentration in the environment. Adaptation of *K*_*d*_ to the mean has been observed in olfactory receptor neurons of the fruitfly [32–34], and might serve to increase the amount of information that the fly olfactory system can encode about its location in a turbulent environment.

### Implications for odor navigation algorithms

With regard to odor navigation algorithms, we note that these fall into two categories: those that rely on local cues (*e.g.* comparison of concentration differences in two sensors [35], comparison of sample arrival times in two sensors [13], the combination of local anemotactic and olfactory cues [36, 37]), and those algorithms that construct a cognitive map (like *infotaxis* [1] and *mapless* [2]). We do not intend to argue for one kind of strategy over the other, but rather to identify aspects of the odor navigation problem that apply to both, as both begin with the acquisition of sensory samples. Our work suggests that these algorithms can operate on a coarse representation of odor concentration since we find that a four-bit representation of the odor intensity reveals almost the same amount of information as finer odor concentration representation. We also found that sampling with two sensors adds substantially to the amount of information about location, and this improvement is not just due to obtaining two samples, but by comparing them in a labelled fashion (as observed in the second row of Fig 3). While this is directly exploited by comparison algorithms using two sensors, we suggest that, navigation algorithms that use an internal model of the odor distribution like *infotaxis* and *mapless* could also be improved by incorporating measurements from two sensors.

Finally, an important caveat of our study is that animals have multi-sensory cues available; here we only consider the single modality of odor and do not integrate information of other modalities, *e.g* visual or mechanosensory flow information, that navigators have access to. In particular, it is crucial for moths and fruitflies to combine flow information via mechanosensory input when walking and visual input when flying for successful navigation [38–41]. For example, since the wind direction may meander substantially, a simple upwind movement can lead a navigator out of the odor plume [8, 42]. Simultaneously recording flow and odor concentration, and analysis along the lines undertaken here, may shed light on useful sampling strategies for combining both sources of information.

### Conclusion

Determining the location of an odor source based on olfactory cues is a challenging problem. We focused on how to optimally sample from the odor distribution when the goal is to determine location with respect to the source. This study shows that the sampling strategy that maximizes information about location under finite resources utilizes two sensors, allowing for the comparison of spatially separated samples, while representing odor concentration in no more than three to four bits. Furthermore, temporal sequences of samples can be averaged to preserve resources while only minimally affecting the amount of information that the sequence conveys.

## Supporting information

S1 Fig Probability distributions of concentrations at the sampling grids (only upper half of locations shown). Columns (*A*), (*B*) and (*C*) correspond to the three conditions *fast flow*, *slow flow* and *boundary flow*. Each row shows log probability distributions at four of the grid points (as indicated by the colors in the inset, top two rows of the figure for the *narrow grid* and bottom two rows for the *wide grid*).
(TIF)

S2 Fig Optimal binarization threshold and bin boundaries for histogram equalization (up to 8 bits). The optimal binarization threshold is shown at the bottom of each panel and is labelled 1*; above it are the bin boundaries of histogram equalization for up to 8 bits (256 bins). Each of the 49 grid placements contributes one sample per bin boundary. Blue corresponds to the *narrow grid* and green corresponds to the *wide grid* of sampling locations. Columns (*A*), (*B*) and (*C*) correspond to the three different conditions *fast flow*, *slow flow* and *boundary flow* respectively. Note that optimal binarization threshold (row labelled 1*) is higher than the histogram-equalization cutpoint (row labelled 1) in all cases except (*C*1), the *narrow grid* *boundary flow* condition.
(TIF)

S3 FigMutual information between location (L) and measurement (M) for different encoding strategies using a “greedy” strategy to allocate bin boundaries.Blue curves correspond to mutual information for the *narrow grid* and green curves correspond to calculations for the *wide grid*. Solid curves represent locations as shown in Fig 1 and shaded curves represent jittered locations. In *A*2 − *C*2, solid lines show information, using knowledge of which sample occurs at which sensor, dashed lines show information ignoring which of two sensors measures which sample.(TIF)

S4 FigMutual information between location (L) and measurement (M) for encoding strategies S(n;1,2) where the time between samples is *τ*_80_ for each condition as indicated by the arrow in Fig 6.Color code as in S3 Fig.(TIF)

S5 Fig Mutual information estimates for sampling at two sensors, as a function of the spacing between them. The spacing used for all two sensor calculations in the main body is shown as solid lines (regular spacing 2.96 mm), double spacing (5.92 mm) as dashed lines and half spacing (1.48 mm) as dotted lines. Top row shows mutual information using knowledge of which sample occurs in which sensor, bottom row shows mutual information neglecting sensor identity. Conditions: *fast flow A*, *slow flow B* and *boundary flow C*.
(TIF)

S6 Fig Mutual information estimates with smaller subsets of the data. The abscissa is the reciprocal of the mutual information estimates over inverse number of samples per location. Panels *A*, *B* and *C* correspond to the conditions *fast flow*, *slow flow* and *boundary flow*; coding strategy is indicated at the top of each panel. Hollow blue and green circles represent mutual information for the narrow- and *wide grid*. Solid lines represent least-squares fits. The intercept with the ordinate represents the extrapolation of mutual information to the limit of infinite data.
(TIF)
