# Supplementary figures and images for: Information-theoretic analysis of realistic odor plumes: What cues are useful for determining location?
Source: PLoS Comput Biol. 2018 Jul 10;14(7):e1006275. doi: 10.1371/journal.pcbi.1006275 (PMC6054425; doi:10.1371/journal.pcbi.1006275)

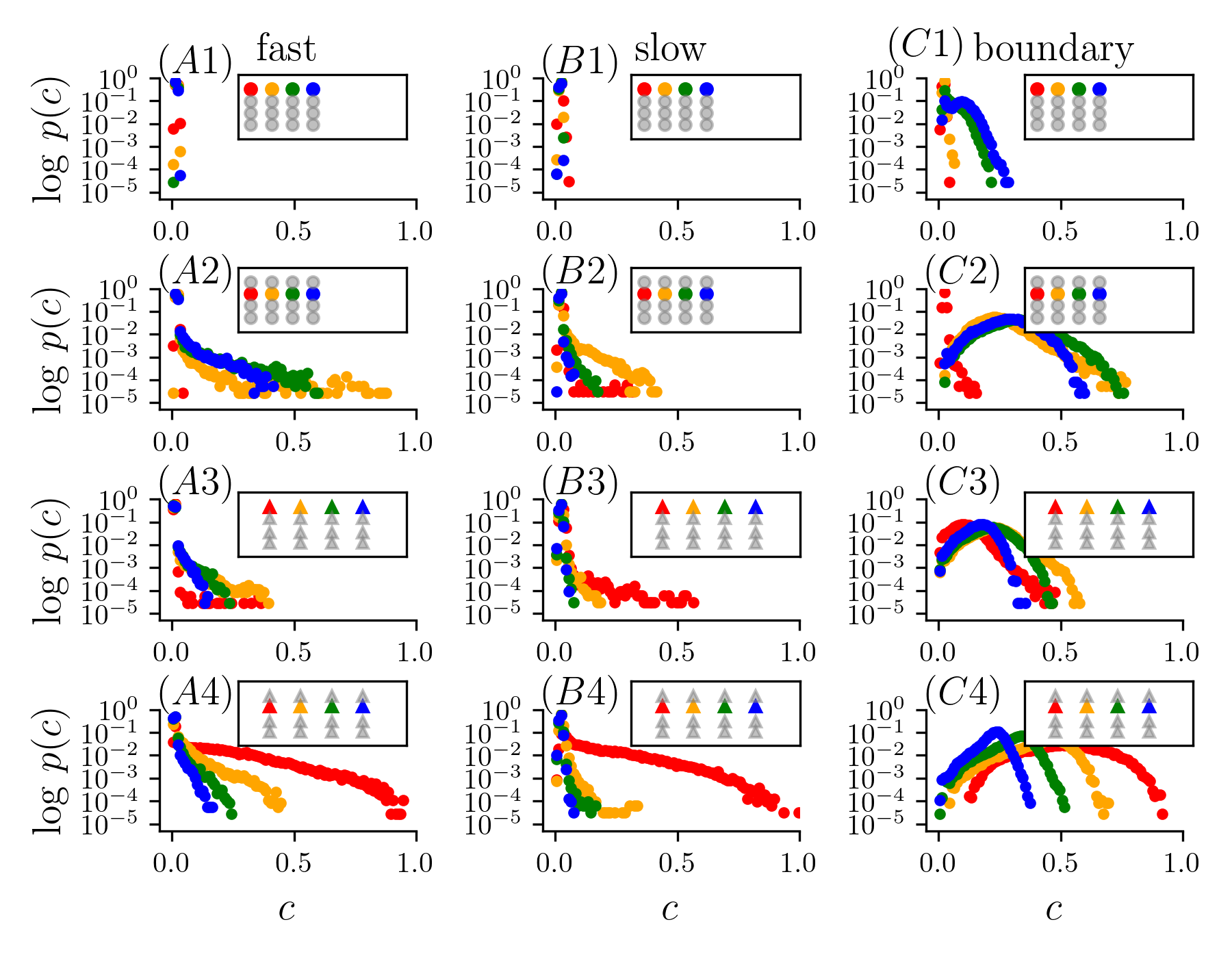

Supplement: S1 Fig — Columns (A), (B) and (C) correspond to the three conditions fast flow, slow flow and boundary flow. Each row shows log probability distributions at four of the grid points (as indicated by the colors in the inset, top two rows of the figure for the narrow grid and bottom two rows for the wide grid). (TIF) [file pcbi.1006275.s001.tif]

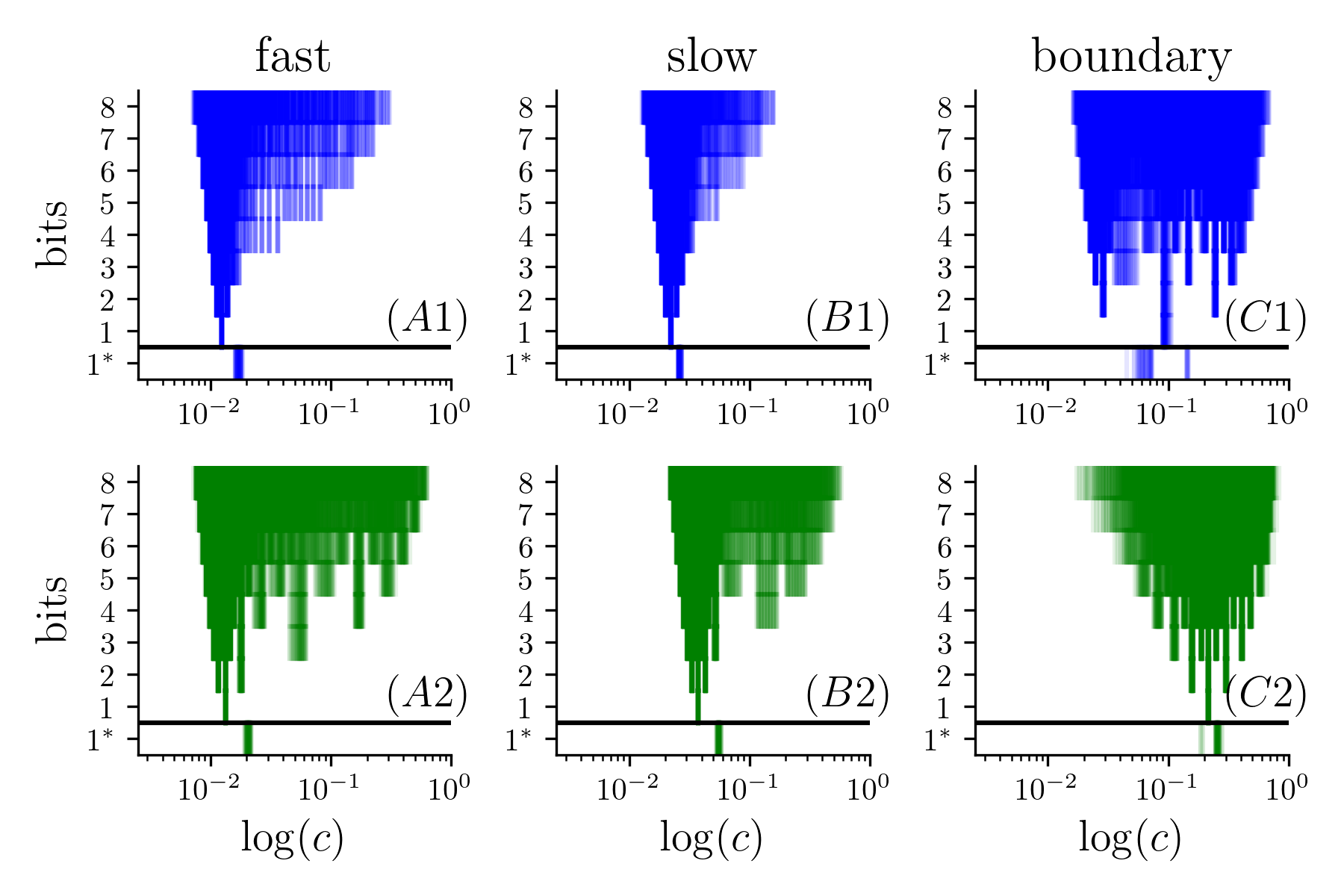

Supplement: S2 Fig — The optimal binarization threshold is shown at the bottom of each panel and is labelled 1*; above it are the bin boundaries of histogram equalization for up to 8 bits (256 bins). Each of the 49 grid placements contributes one sample per bin boundary. Blue corresponds to the narrow grid and green corresponds to the wide grid of sampling locations. Columns (A), (B) and (C) correspond to the three different conditions fast flow, slow flow and boundary flow respectively. Note that optimal binarization threshold (row labelled 1*) is higher than the histogram-equalization cutpoint (row labelled 1) in all cases except (C1), the narrow grid boundary flow condition. (TIF) [file pcbi.1006275.s002.tif]

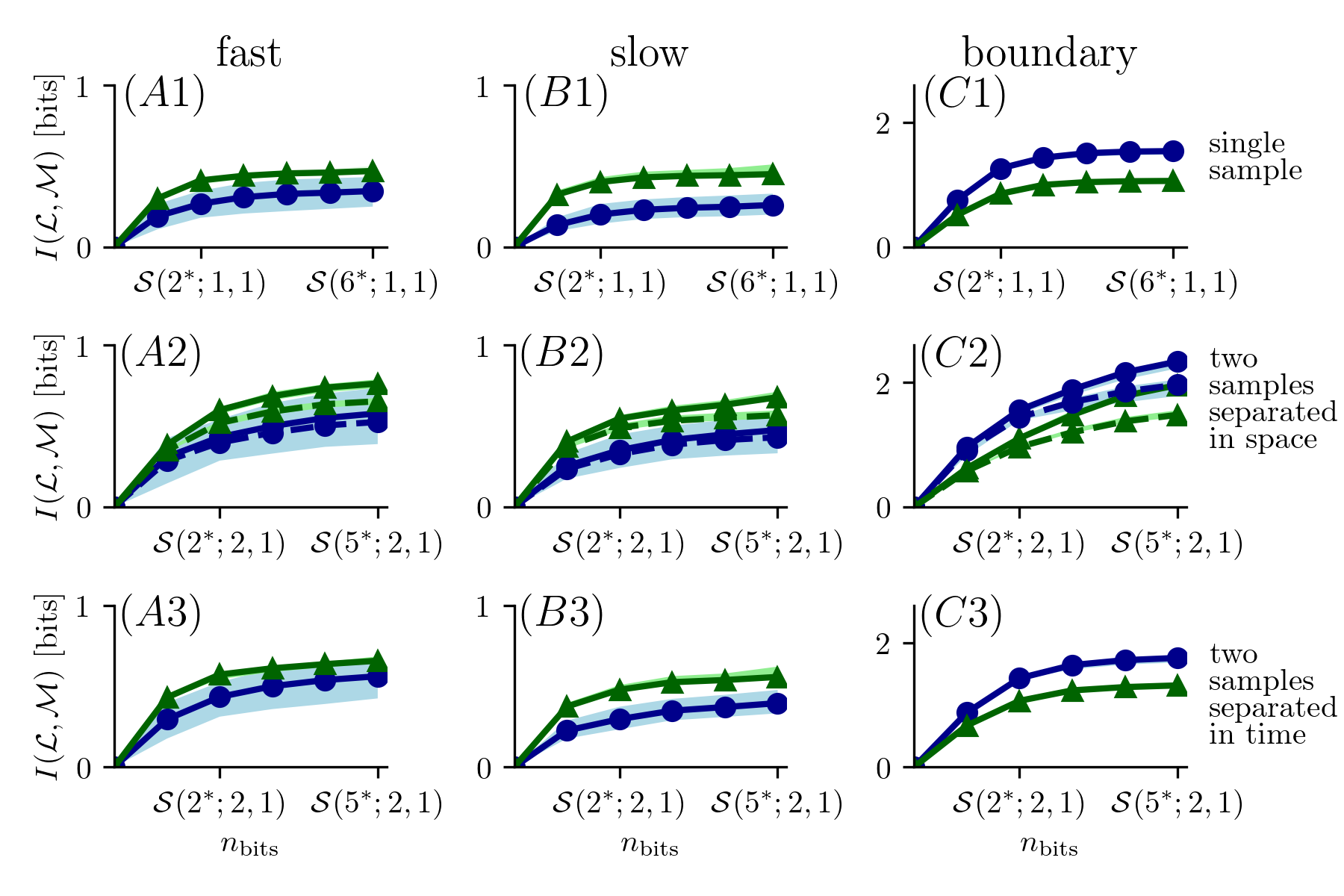

Supplement: S3 Fig — Blue curves correspond to mutual information for the narrow grid and green curves correspond to calculations for the wide grid. Solid curves represent locations as shown in Fig 1 and shaded curves represent jittered locations. In A2 − C2, solid lines show information, using knowledge of which sample occurs at which sensor, dashed lines show information ignoring which of two sensors measures which sample. (TIF) [file pcbi.1006275.s003.tif]

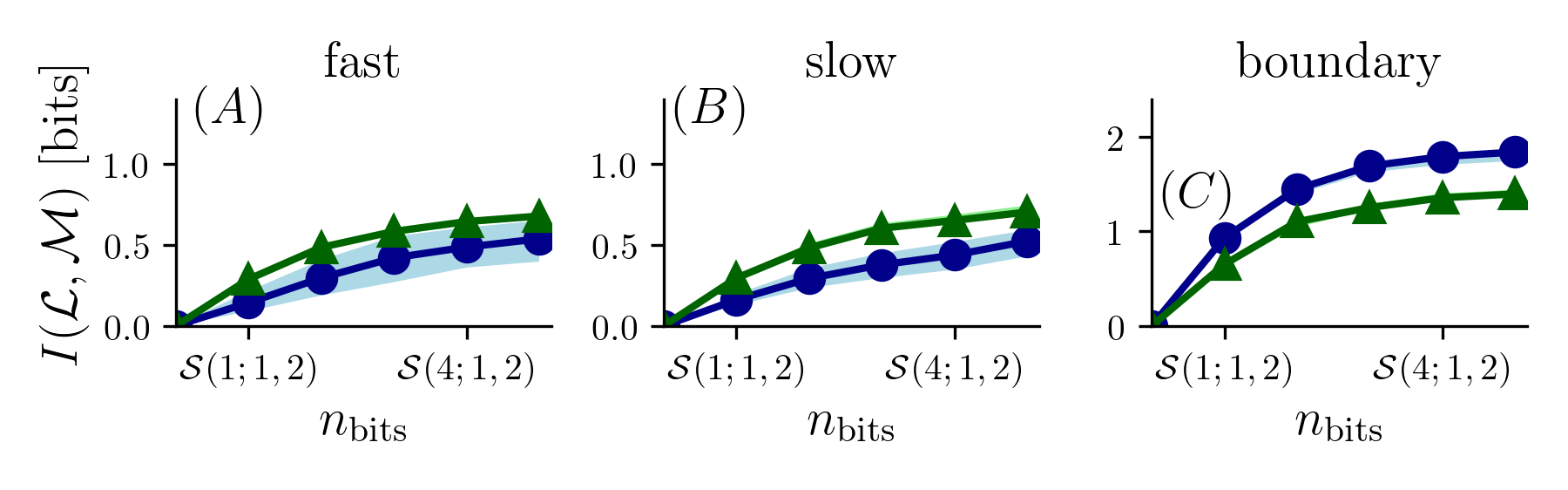

Supplement: S4 Fig — Color code as in S3 Fig. (TIF) [file pcbi.1006275.s004.tif]

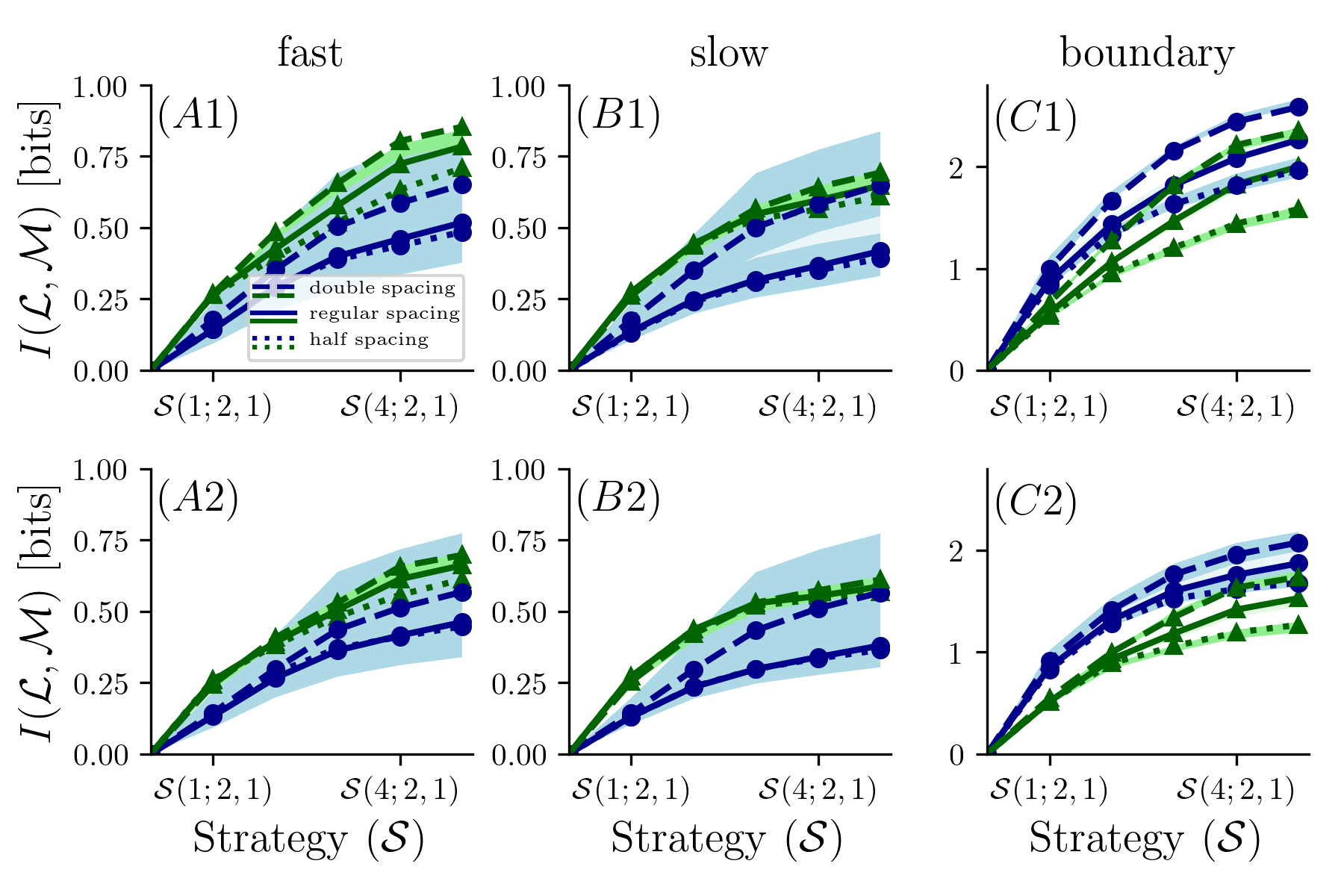

Supplement: S5 Fig — The spacing used for all two sensor calculations in the main body is shown as solid lines (regular spacing 2.96 mm), double spacing (5.92 mm) as dashed lines and half spacing (1.48 mm) as dotted lines. Top row shows mutual information using knowledge of which sample occurs in which sensor, bottom row shows mutual information neglecting sensor identity. Conditions: fast flow A, slow flow B and boundary flow C. (TIF) [file pcbi.1006275.s005.tif]

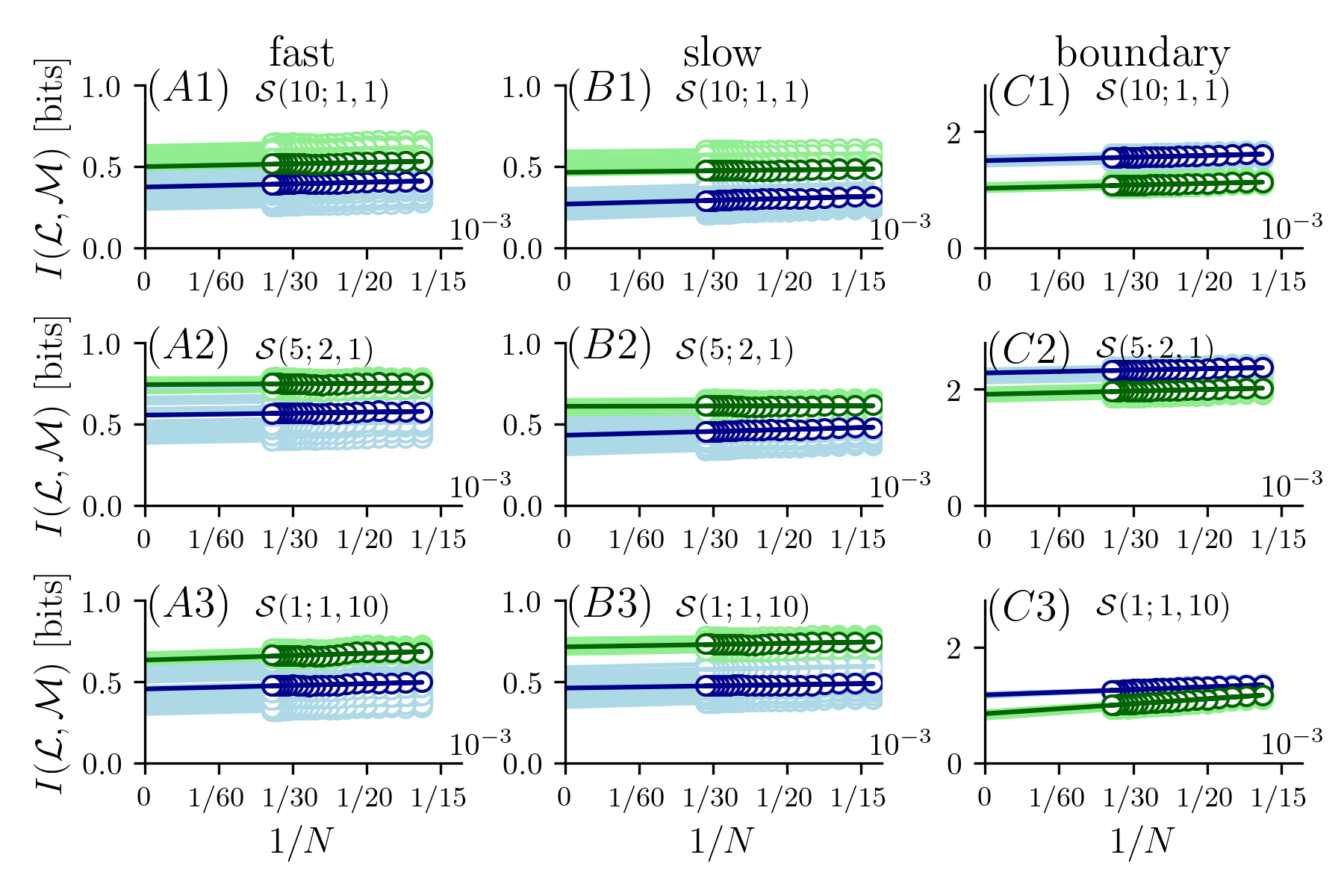

Supplement: S6 Fig — The abscissa is the reciprocal of the mutual information estimates over inverse number of samples per location. Panels A, B and C correspond to the conditions fast flow, slow flow and boundary flow; coding strategy is indicated at the top of each panel. Hollow blue and green circles represent mutual information for the narrow- and wide grid. Solid lines represent least-squares fits. The intercept with the ordinate represents the extrapolation of mutual information to the limit of infinite data. (TIF) [file pcbi.1006275.s006.tif]
